# Tooth loss early in life induces hippocampal morphology remodeling in senescence-accelerated mouse prone 8 (SAMP8) mice

**DOI:** 10.7150/ijms.40241

**Published:** 2020-02-10

**Authors:** Masahisa Katano, Kyoko Kajimoto, Mitsuo Iinuma, Kagaku Azuma, Kin-ya Kubo

**Affiliations:** 1 Department of Pediatric Dentistry, Asahi University School of Dentistry, 1851 Hozumi, Mizuho, Gifu, 501-0296, Japan; 2 Department of Anatomy, School of Medicine, University of Occupational and Environmental Health, 1-1 Iseigaoka, Yahatanishi-ku, Kitakyushu, Fukuoka, 807-8555, Japan; 3 Graduate School of Human Life Science, Nagoya Women's University, 3-40 Shioji-cho, Mizuho-ku, Nagoya, Aichi, 467-8610, Japan

**Keywords:** tooth loss, hippocampus, transmission electron microscopy, mitochondria, myelin sheath, synapse

## Abstract

Long-term tooth loss is associated with the suppression of hippocampal neurogenesis and impairment of hippocampus-dependent cognition with aging. The morphologic basis of the hippocampal alterations, however, remains unclear. In the present study, we investigated whether tooth loss early in life affects the hippocampal ultrastructure in senescence-accelerated mouse prone 8 (SAMP8) mice, using transmission electron microscopy. Male SAMP8 mice were randomized into control or tooth-loss groups. All maxillary molar teeth were removed at 1 month of age. Hippocampal morphologic alterations were evaluated at 9 months of age. Tooth loss early in life induced mitochondrial damage and lipofuscin accumulation in the hippocampal neurons. A thinner myelin sheath and decreased postsynaptic density length were also observed. Our results revealed that tooth loss early in life may lead to hippocampal ultrastructure remodeling and subsequent hippocampus-dependent cognitive impairment in SAMP8 mice with aging.

## Introduction

The rapid growth of elderly populations is associated with an increase in the number of people with senile dementia throughout the world. Currently, 47 million patients have dementia and the number will increase to 75 million globally by 2030. Dementia is a major health and socioeconomic problem 1. Understanding the pathogenesis of dementia will facilitate the development of novel therapeutic strategies.

Numerous studies indicate an association between masticatory dysfunction and cognitive impairment 2-4. Teeth are important for mastication. Masticatory activity is crucial for overall nutrition, general health, and hippocampus-dependent cognition 4, 5. The prevalence of tooth loss increases with age. Impaired masticatory function induced by tooth loss is a risk factor for cognitive impairment, such as dementia and Alzheimer's disease 2, 3, 6. Previous studies revealed that long-term tooth loss, representing a chronic psychologic stressor, induces stress reactions, activates the hypothalamic-pituitary-adrenal axis, and stimulates the adrenal cortex to secrete excess glucocorticoids, corticosterone in rodents and cortisol in humans 7-10. Animal experiments demonstrate that the blood corticosterone concentration in animals with long-term tooth loss is significantly higher than that in controls 11, 12. The lipophilic nature of glucocorticoids facilitates their rapid entry into the brain. The hippocampus contains the highest level of glucocorticoid receptors, making it a target of stress hormone effects 5, 13. Mice and rats with tooth loss exhibit impaired hippocampal neurogenesis as well as impaired spatial learning and memory in the Morris water maze test 12, 14-16. The effect of early tooth loss on the hippocampal morphology during aging, however, is largely unknown. Identifying the effects of long-term tooth loss on hippocampal structural remodeling will improve our understanding of the relationship between masticatory activity and hippocampus-dependent cognition in the elderly.

The present study examined the effects of tooth loss early in life on the hippocampal ultrastructure in senescence-accelerated mouse strain P8 (SAMP8) mice. SAMP8 mice exhibit age-related behavioral and morphologic alterations, as well as spatial learning deficits 12, 17 . The SAMP8 mouse is a murine model of senile dementia 18. In order to explore the underlying mechanisms of the senile dementia, we examined the influence of long-term tooth loss on hippocampal morphologic remodeling in aged SAMP8 mice.

## Materials and methods

### Animals and experimental design

SAMP8 mice obtained from Japan SLC (Hamamatsu, Shizuoka, Japan) were used. Mice were maintained on standard rodent pellet chow (CE-2, CLEA Japan, Inc., Tokyo, Japan) available ad libitum. Mice were housed in plastic cages under controlled temperature and humidity (23 ± 1°C, 55 ± 5%) with a 12-h light-dark cycle. The experimental protocol complied with the guidelines for laboratory animal care and use of Asahi University. Mice were randomly divided into control and tooth-loss groups (n=7). Extraction of the maxillary molar teeth was performed as previously described 7, 11, 12, 19. Briefly, 1-month-old male mice were anesthetized with sodium pentobarbital and all maxillary molar teeth were removed using dental tweezers. The development of mouse teeth continues up to postnatal day 25 20. To ensure complete tooth removal, we extracted the molar teeth at 1 month of age. Control mice underwent the same anesthesia procedures, but their maxillary molar teeth were not removed. After surgery, the animals were raised under standard conditions for 8 months. The average daily food intake and body weights were measured once per month.

### Blood corticosterone analysis

At 9 months of age, all mice were injected intraperitoneally with an overdose of sodium pentobarbital. The blood was extracted at the beginning of the dark cycle around 20:00, when the mouse blood corticosterone level is highest 16. The serum corticosterone concentration was measured using a mouse corticosterone enzyme-linked immunosorbent assay kit (AssayPro, Saint Charles, MO, USA), as previously reported 21, 22.

### Light microscopy

Mice were given an overdose of sodium pentobarbital and perfused with 0.9% sodium chloride, followed by 4.0% paraformaldehyde through the aorta. The brains were carefully removed and immersed further in the same fixative overnight at 4℃. The brains were coronally sectioned at 10 μm using a cryostat. Sections were stained cresyl violet for histological observation.

### Transmission electron microscopy

Mice were perfused through the aorta with 0.9% sodium chloride, followed by 2% paraformaldehyde and 2.5% glutaraldehyde in 0.1 M phosphate buffer, pH 7.4. The brains were dissected and removed from the skulls quickly and carefully, and immersed further in the same fixative overnight at 4℃. After rinsing with phosphate buffer, the brain tissues were then post-fixed with 1% aqueous osmium tetroxide solution for 1 h. The tissues were then dehydrated by passing through a graded series of acetone. Specimens were embedded using epoxy resin. The ultra-thin sections were prepared with a diamond knife on a Porter-Blum MT-1 ultramicrotome (Ivan Sorvall, Inc., Norwalk, CT, USA), stained with uranyl acetate and lead salts, and observed under a transmission electron microscope (JEM-1400Plus, JEOL, Tokyo, Japan).

The areas of the cytoplasm, mitochondria, and lipofuscin in the pyramidal neurons of the hippocampal CA3 region were determined using imaging software (CellSens, Olympus Corporation, Tokyo, Japan). Ten images per animal were selected and the volume density of the cytoplasm occupied by mitochondria and lipofuscin was estimated. The volume density of mitochondria and lipofuscin was determined according to standard stereological principles described previously 23. The number of lipofuscin granules per neuron and the number of microtubules per axon were also calculated. Myelin structures in the CA3 region were quantitatively analyzed in 10 images per animal, which in total contained 210 axons. The G-ratio of the inner axonal diameter to the outer diameter was determined on the same myelin axonal structures as described previously 24, 25. We identified synapses through the existence of synaptic vesicles within the presynaptic terminals and the postsynaptic density (PSD) in the postsynaptic elements. Measurement of PSD length was carried out using a previously described method 25, 26. Fifty synapses per animal were counted.

### Statistical Analysis

All values are reported as means ± standard deviation (SD). Statistical analysis was performed using SPSS version 22. Differences between the control and tooth-loss groups were evaluated using an unpaired t-test. Differences were considered statistically significant at p < 0.05.

## Results

### Daily food intake, body weight and serum corticosterone levels

**Figure 1:**
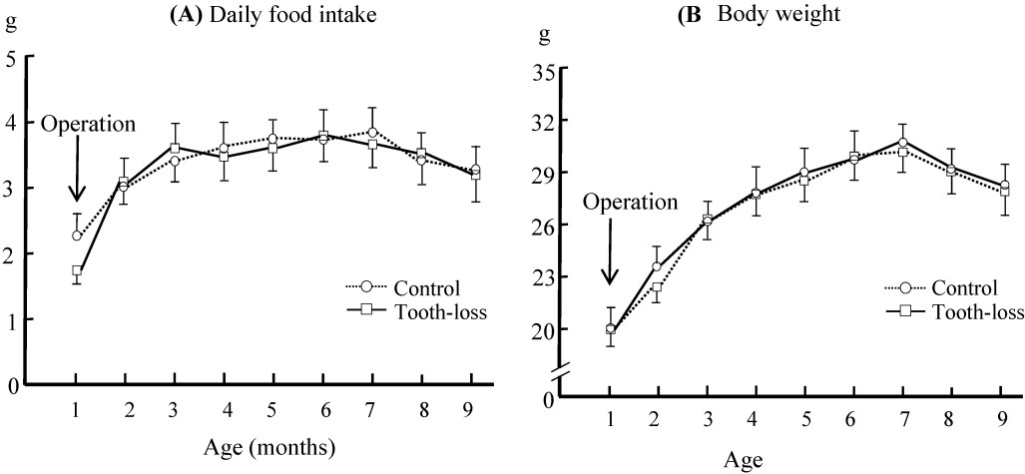
** The daily food intake (A) and body weight (B) in the control and tooth-loss mice.** There were no significant differences between the control and tooth-loss mice throughout the experimental period.

The time course of the average daily food intake and body weight in control and tooth-loss group is shown in Fig. 1. The food intake and body weight of the tooth-loss group tended to decrease for a few days after the extraction and then returned to the preoperative levels. There were no significant differences between the control and tooth-loss groups throughout the experimental period (Fig. 1).

**Figure 2:**
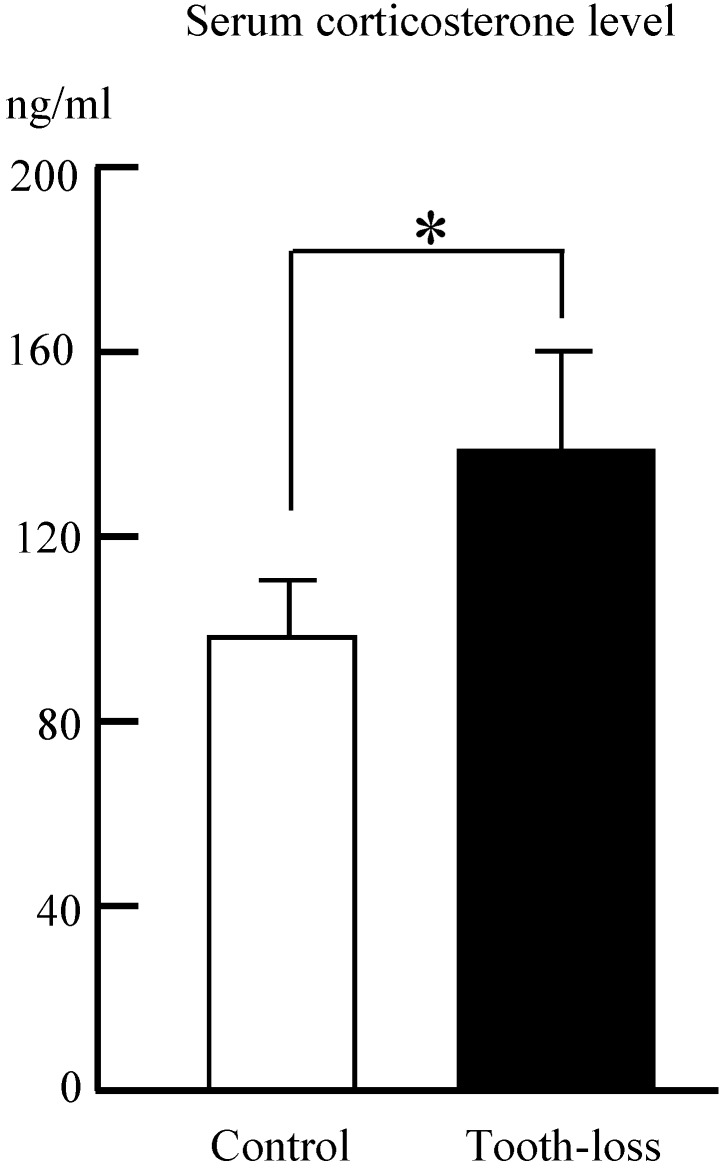
** The serum corticosterone level in the control and tooth-loss mice.**The serum corticosterone level was significantly higher in the tooth-loss mice than in the control mice. *p < 0.05.

The serum corticosterone levels in both the control and tooth-loss groups are shown in Fig. 2. The serum corticosterone level was significantly higher in the tooth-loss group than in the control group (Fig. 2).

### Morphologic features of the hippocampal neurons

**Figure 3:**
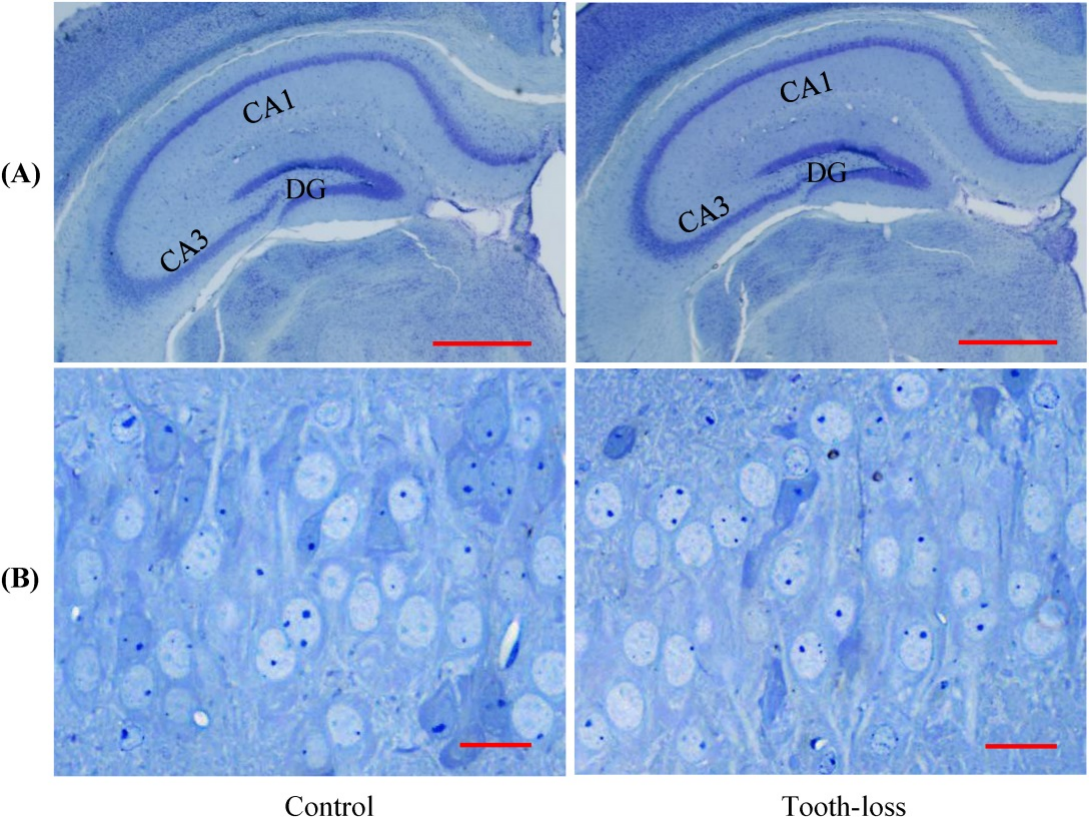
** Light micrographs of the mouse hippocampus (A).** The general morphology of the dentate gyrus (DG) and cornu ammonis (CA1, CA3) regions is similar between the control and tooth-loss mice. Scale bars: 1 mm. **Light micrographs of the CA3 pyramidal cell layer (B).** There are no observable morphological differences in the hippocampal CA3 pyramidal cells between the control and tooth-loss mice. Scale bars: 20 μm.

**Figure 4:**
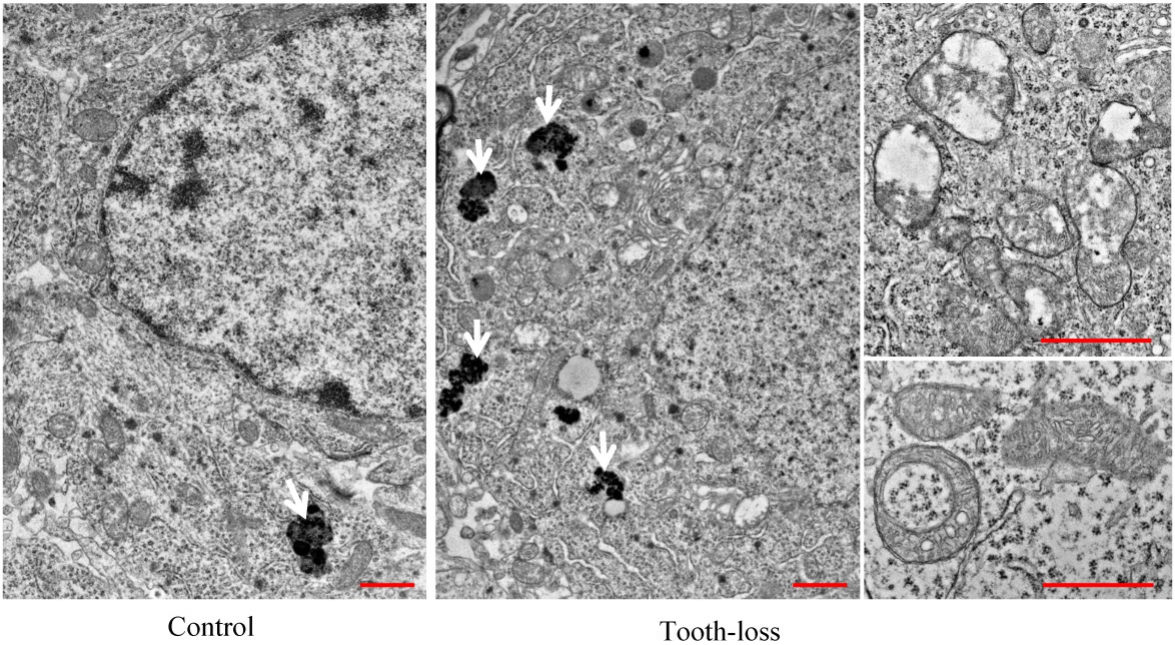
** The ultrastructural features of the mouse hippocampal neurons.** There were many mitochondria with intact cristae and few lipofuscins in the hippocampal neurons of the control mice. Numerous lipofuscins, swollen and ring-shaped mitochondria were observed in the tooth-loss mice. Arrows: lipofuscins. Scale bars: 1 μm.

**Figure 5:**
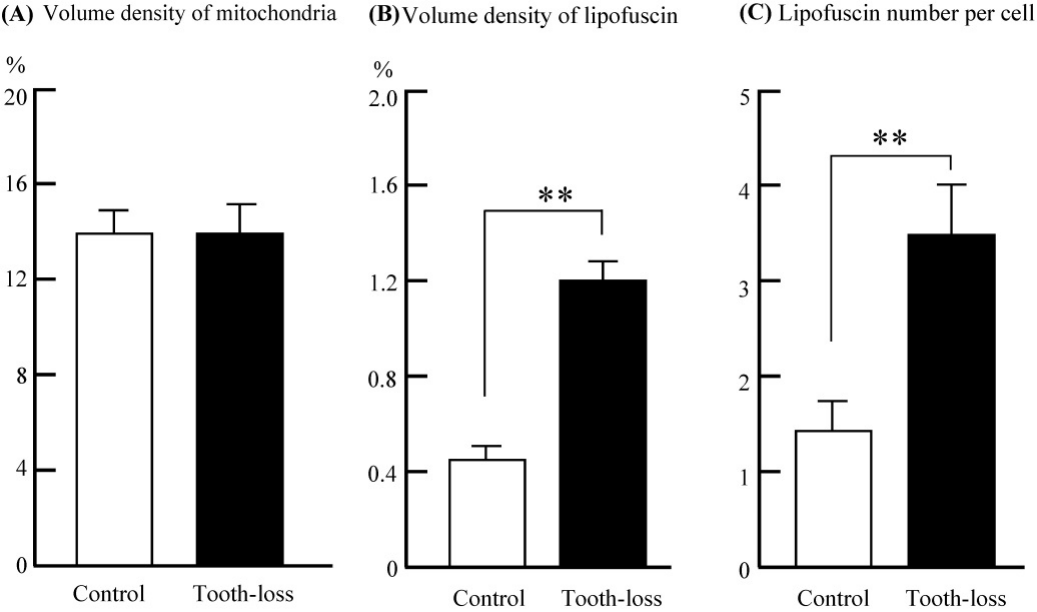
** Volume density of mitochondria (A) and lipofuscin (B) in the cytoplasm, and the average lipofuscin number (C) per neuron of the mouse hippocampus.** There was no significant difference in the volume density of mitochondria between the control and tooth-loss mice. Compared with the control group, the volume density and number of lipofuscin granules were significantly higher in the tooth-loss mice. **p < 0.01.

The general morphology of the hippocampal cornu ammonis (CA1, CA3) regions and the dentate gyrus (DG) was similar between the control and tooth-loss groups (Fig. 3A). Cresyl violet staining revealed that there were no observable morphological differences in the hippocampal CA3 pyramidal cells between the control and tooth-loss groups (Fig. 3B). Neuronal polarity was preserved in the tooth-loss group. The rough endoplasmic reticulum, Golgi complex and mitochondria were well-developed in both groups. We observed numerous swollen mitochondria, missing cristae, and ring-shaped mitochondria in the tooth-loss group (Fig. 4). There was no significant difference in the volume density of mitochondria between the control and tooth-loss groups (Fig. 5A). The rough endoplasmic reticulum and Golgi complex exhibited no marked difference between the control and tooth-loss groups.

Lipofuscin is an irregularly shaped structure with varying degrees of electron density. Lipofuscin granules are scattered in the cytoplasm. We observed few lipofuscin granules in the hippocampal neurons of the control mice. Several lipofuscin deposits, however, were observed in the hippocampal neurons of the tooth-loss group (Fig. 4). We calculated the volume density and number of lipofuscin granules per neuron. Compared with the control group, the volume density and number of the lipofuscin granules were significantly higher in the tooth-loss group (Fig. 5B, C).

**Figure 6:**
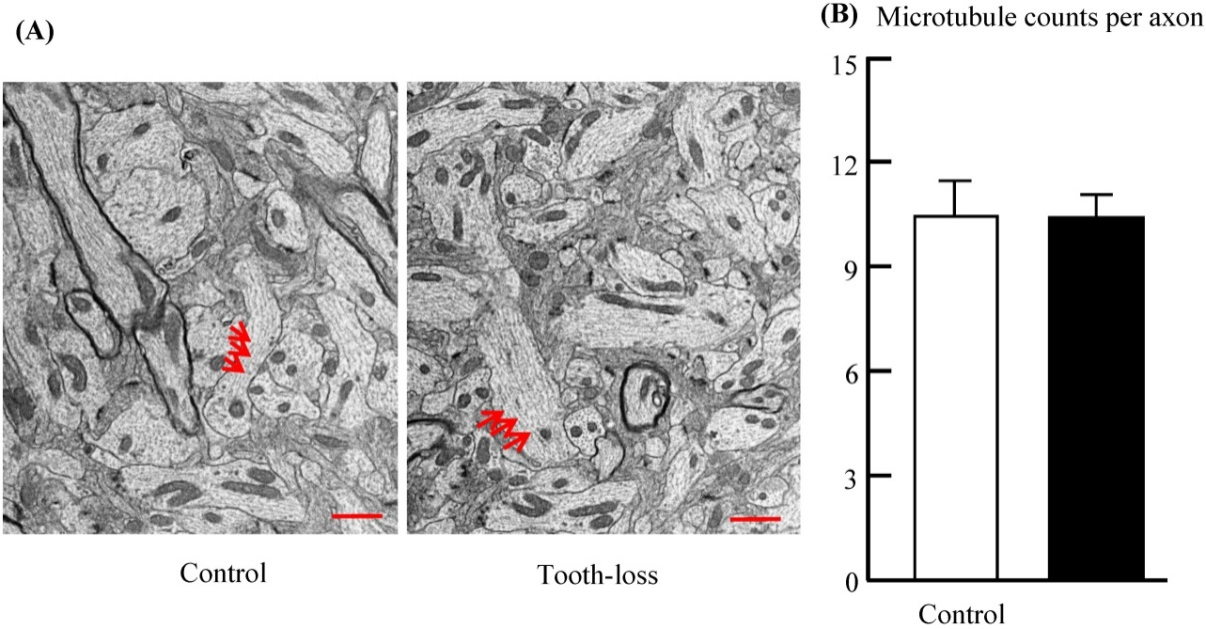
** Electron micrographs of the mouse hippocampal microtubules (A) and the average number of microtubules per axon (B).** Axons were filled with numerous microtubules arranged in parallel. The morphologic features of the microtubules are similar in the control and tooth-loss mice. Quantitative analysis revealed that there was no significant difference in the number of microtubules between the control and tooth-loss mice. Arrows: microtubules. Scale bars: 1 μm.

Microtubules play a critical role in transporting neurotransmitters within the axons of target neurons. Axons were filled with numerous microtubules arranged in parallel (Fig. 6). The morphologic features of the microtubules, such as microtubule number, length, orientation, and distribution, were similar between groups (Fig. 6A). Quantitative analysis revealed that there was no significant difference in the number of microtubules between the control and tooth-loss groups (Fig. 6B).

### Myelin sheath and PSD length

**Figure 7:**
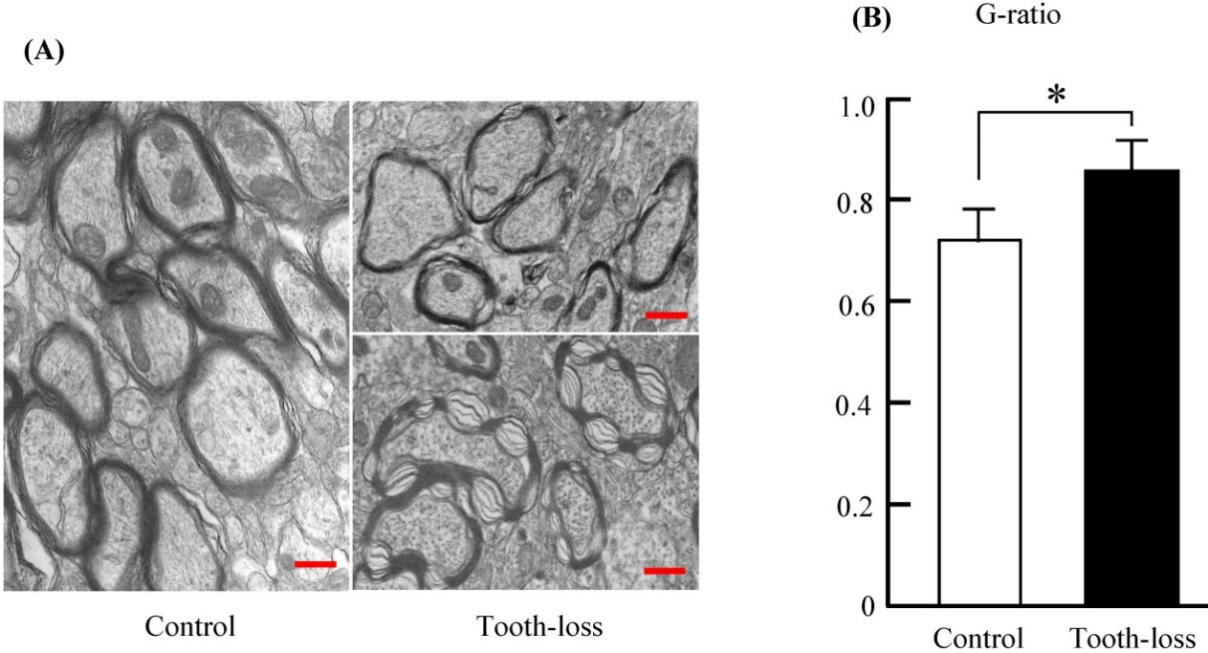
** Electron micrographs of the mouse hippocampal myelin sheath (A) and the G-ratio (B).** The myelin sheaths were structured loosely and disordered in texture in the tooth-loss mice. The G-ratio was significantly higher in the tooth-loss mice, revealing a thinner myelin sheath. Scale bars: 1 μm. *p < 0.05.

Myelinated fibers in the central nervous system are essential for neuronal communication and are involved in neurotransmission 27. The thickness of the myelin sheath is a determinant of neuronal conduction velocity 28. Myelinated nerve fibers in the control mice appeared normal, with a concentric dense multi-lamellar structure (Fig. 7). There were numerous irregular myelin sheaths in the tooth-loss mice (Fig. 7A). Myelin sheaths were structured loosely and disordered in texture. Some myelin sheaths in the tooth-loss mice had a collapsed, disrupted, and disordered configuration. Quantitative analysis showed that the G-ratio was significantly higher in the tooth-loss mice than in the control mice, revealing a thinner myelin sheath in the tooth-loss mice (Fig. 7B).

**Figure 8:**
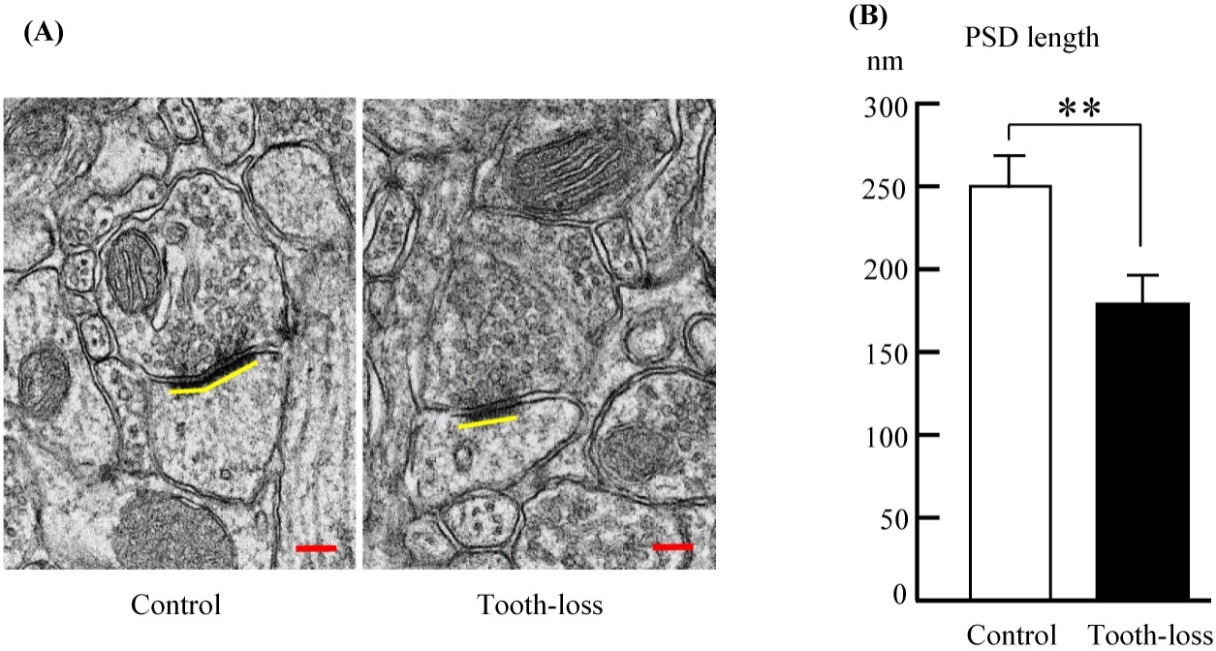
** Electron micrographs of the mouse hippocampal synapses (A) and the PSD length (B).** Compared with the control mice, the average PSD length was significantly shorter in the tooth-loss mice. Scale bars: 100 nm. **p < 0.01.

Alterations of synapse size are linked to neurodevelopmental disorders 29. We determined the synapse size by measuring the PSD length, which may be associated with alterations in synaptic efficacy, synaptic plasticity, and intellectual disability 26, 30, 31. A representative ultrastructure of the hippocampal synapses in SAMP8 mice is shown in Figures 6. Compared with the control mice, the average PSD length was significantly shorter in tooth-loss mice (Fig. 8A, B).

## Discussion

This is the first study to investigate hippocampal ultrastructural changes induced by long-term tooth loss in aged SAMP8 mice. In the present study, we found that the main characteristics of the ultrastructural remodeling in hippocampus of tooth-loss mice were mitochondrial damage, lipofuscin accumulation, thinning of the myelin sheath and shortening of postsynaptic density. These findings indicate that hippocampal structural remodeling can be induced by tooth loss early in life.

Previous studies showed that tooth loss early in life induced an increase in the corticosterone level, consistent with the present finding. Tooth loss early in life reduces spine density and synaptophysin expression in the hippocampus, and leads to impaired hippocampus-dependent cognition with aging 7, 10, 12, 14.

The adult hippocampal neurogenesis is important for learning and memory 11, 12, 32. Immuno fluorescence study demonstrated that tooth loss inhibited the hippocampal neurogenesis both in the normal CD-1 mice and SAMP8 mice 11, 12, 32. Brain-derived neurotrophic factor (BDNF) is a member of the neurotrophin family, which is widely expressed in the brain. The hippocampal BDNF levels are involved in the regulation of the hippocampal neurogenesis and synaptic plasticity 11, 33. Animal studies indicated that tooth loss decreased hippocampal BDNF expression levels both in C57BL/6J and SAMP8 mice 11, 33. We consider that the effect of tooth loss on the hippocampus was similar for genetically different inbred murine strains.

There is no consensus on the effects of tooth loss on body weight. Kawahata et al. extracted molar teeth at 8 weeks of SAMP8 mice and found the body weights decreased significantly from 10 to 24 weeks after surgery 10. Several other studies, however, demonstrated that tooth loss in SAMP8 mice may lead to temporary weight reduction immediately after tooth loss, with no significant change in body weight thereafter 12, 16, 34. In the present study, we observed that after surgery, the daily food intake and body weight of the tooth-loss group tended to decrease compared with that of the control group. However, there were no significant differences between the two groups throughout the study.

Mitochondrial dysfunction is a common characteristic of aging and neurodegenerative disorders 35, 36. Improvement of mitochondrial function could be a strategy to slow aging and age-related neurodegenerative diseases. In the present study, we identified the presence of abnormal mitochondria in the hippocampus of tooth-loss mice. The morphology of other organelles, including the rough endoplasmic reticulum and Golgi complex, as well as the plasma membrane remained unaltered in the hippocampal neurons of tooth-loss mice, suggesting that mitochondria is more vulnerable to damage caused by tooth loss.

Lipofuscin deposits, as a marker of aging, are often observed in hippocampal neurons of the aged animals 37, 38. Lipofuscin deposits are observed in the hippocampal neurons of aged SAMP8 mice, particularly after kainite administration. Senile SAMP8 mice are highly vulnerable to oxidative stress. Kainite is considered to induce oxidative damage, which might contribute to neuronal lipofuscin accumulation. The accumulation of lipofuscin granules is considered an oxidative stress response. The present study showed that the volume density and number of lipofuscin granules were significantly higher in tooth-loss mice than in control mice. We consider that long-term tooth loss induces chronic stress, and accelerates the hippocampal neuronal aging process, due to the accumulation of lipofuscin granules.

The mitochondrion is an important organelle for maintaining normal cell function, and plays a main role in the response to oxidative stress 39. Mitochondrial dysfunction could cause the overproduction of reactive oxygen species, resulting in oxidative stress. As the formation of lipofuscin is involved in oxidative stress, lipofuscin accumulation might be associated with mitochondrial damage. In this study, we observed many abnormal mitochondria, including those without cristae, swollen mitochondria, and ring-shaped mitochondria in the hippocampal neurons of long-term tooth-loss mice. While the morphology of the rough endoplasmic reticulum, Golgi complex, and cytoplasmic membrane remained intact. These findings suggest that the mitochondria are particularly vulnerable to oxidative damage due to long-term tooth loss in aged SAMP8 mice.

The central myelin sheaths contribute to the conduction of neuronal impulses in myelinated fibers. Abnormal myelin sheaths, including thinning, collapse, disruption, as well as disordered arrangement of myelin sheaths could result in decreased neuronal conduction 24. Structural alterations of hippocampal myelin sheaths are associated with slowed impulse conduction between the hippocampus and other brain regions, triggering hippocampus-dependent cognitive impairment 25, 40. Like mitochondria, myelin sheath is selectively vulnerable to oxidative stress 41. The central myelin sheaths are produced by oligodendrocytes. We previously demonstrated that stress exposure affects the hippocampal oligodendrocytes and myelin sheaths 25. We speculate that the abnormal myelin sheaths are linked to oligodendrocyte degeneration in long-term tooth-loss SAMP8 mice, although we did not evaluate oligodendrocytes in this study.

Synapses are crucial for transmitting nerve signals. Synaptic plasticity plays an essential role in learning and memory 42. The PSD is a complex molecular assembly below the postsynaptic membrane. Induction of long-term potentiation is related to increases in PSD length 43. PSD length is a crucial parameter for synaptic plasticity and neurobehavioral assessment 30. We found that long-term tooth loss shortened the hippocampal PSD length, and impaired synaptic plasticity, leading to hippocampus-dependent learning deficits.

Microtubules comprise a major component of the neuronal cytoskeleton, which plays a critical role in facilitating long-distance transport of neurotransmitters to synapses 44. Neurons are highly susceptible to microtubule defects, which occur in various neurodegenerative diseases. Microtubule defects include abnormalities of microtubule number, length, orientation, distribution and bundling. In our work, we observed no morphologic alterations of the microtubules in aged tooth-loss mice. A previous study indicated that chronic social stress decreased the brain microtubule protein network dynamics and polymerization ability in rats 45. Whether long-term tooth loss affects microtubule protein network activity and dynamics needs to be confirmed.

In conclusion, the present findings indicate that tooth loss early in life causes mitochondrial damage and lipofuscin accumulation in the hippocampal neurons, accompanied by a thinner myelin sheath and decreased postsynaptic density length in the hippocampus of the aged SAMP8 mice. These findings demonstrate that tooth loss early in life can lead to hippocampal ultrastructure remodeling, resulting in hippocampus-dependent cognitive impairment in SAMP8 mice with aging.
